# An Effective Chemical Permeabilization of Silkworm Embryos

**DOI:** 10.3390/bioengineering10050563

**Published:** 2023-05-08

**Authors:** David Urbán-Duarte, Shuichiro Tomita, Hiroki Sakai, Hideki Sezutsu, José Fernando De La Torre-Sánchez, Yooichi Kainoh, Seiichi Furukawa, Keiro Uchino

**Affiliations:** 1 Centro Nacional de Recursos Genéticos, Instituto Nacional de Investigaciones Forestales, Agrícolas y Pecuarias, Tepatitlán de Morelos 47600, Jalisco, Mexico; 2 Institute of Agrobiological Sciences, National Agriculture and Food Research Organization, 1-2 Owashi, Tsukuba 305-8634, Ibaraki, Japansakaih786@affrc.go.jp (H.S.); hsezutsu@affrc.go.jp (H.S.); 3 Centro Nacional de Investigación Disciplinaria en Agricultura Familiar, Instituto Nacional de Investigaciones Forestales, Agrícolas y Pecuarias, Ojuelos 47540, Jalisco, Mexico; delatorre.fernando@inifap.gob.mx; 4 Faculty of Life and Environmental Sciences, University of Tsukuba, Tennodai 1-1-1, Tsukuba 305-8572, Ibaraki, Japan; kainoh.yooichi.gf@u.tsukuba.ac.jp (Y.K.); furukawa.seiichi.ew@u.tsukuba.ac.jp (S.F.)

**Keywords:** permeabilization, dechorionated embryo, silkworm, *Bombyx mori*

## Abstract

The lipid layer surrounding the vitelline membrane of insect eggs has a critical role in the waterproofing and desiccation resistance of embryos. However, this lipid layer also prevents the flux of chemicals into the embryos, such as cryoprotectants, which are required for successful cryopreservation. The permeabilization studies of silkworm embryos remain insufficient. Therefore, in this study, we developed a permeabilization method to remove the lipid layer in the silkworm, *Bombyx mori*, and examined factors affecting the viability of dechorionated embryos, including the types and exposure times of chemicals and embryonic stages. Among the chemicals used, hexane and heptane were effective for permeabilization, whereas Triton X-100 and Tween-80 were less effective. Regarding the embryonic stages, there were significant differences between 160 and 166 h after egg laying (AEL) at 25 °C. Consequently, we found that the treatment of 160 AEL embryos with hexane for 30 s was the best condition for the permeability and viability of embryos, in which over 62% of the permeabilized embryos grew up to the second larval instar and their moths could lay fertilized eggs. Our method can be used for various purposes, including permeability investigations using other chemicals and embryonic cryopreservation.

## 1. Introduction

Current advances in genetic technologies have permitted the manipulation of the silkworm *Bombyx mori* genome to produce important strains [1,2,3,4,5,6]. To date, all these strains have been maintained by traditional rearing practices; however, this can result in the loss of strains due to climate change, disease, social change, selection errors, mutations, and disasters. Therefore, the development of a reliable long-term cryopreservation method is critical to avoid the loss of these silkworm strains. Cryopreservation methods for *B. mori* have already been developed using the ovary and sperm [7,8,9,10,11]; however, these methods require recipient individuals to reproduce the progenies. In contrast, cryopreserved fertilized eggs can be reproduced independently. As a result, we aimed to develop a cryopreservation method for *B. mori* embryos.

In insect eggs, the lipid layer that covers the vitelline membrane prevents the flux of water and small molecules into the embryo, playing a key role in waterproofing and desiccation resistance [12]. Lipid layers have been identified in several insect orders, including Heteroptera, Diptera, and Lepidoptera [13,14,15,16,17,18,19,20,21,22]. However, this lipid layer constitutes a barrier to the successful cryopreservation of insect embryos [23]. As a result, the removal of the lipid layer is of great importance to render embryos permeable to water, cryoprotectants, and other molecules. Organic solvents, such as hexane and heptane (alkanes), have been used to remove the lipid layer after dechorionation in dipteran species [18,24,25,26,27,28,29]. For those, the embryo viability and permeability seemed to be dependent on embryonic development. Other chemicals, such as Triton X-100 and Tween-80, have also been suggested for removing the lipid layer from lepidopteran species [28,30]. Information on the types of chemicals used and the exposure times for permeabilization in other species indicated that modified treatments should be developed for silkworm embryos. In addition to permeabilization, there has been an issue with the chorion present in the outermost layer of the eggshell, which is another barrier to successful cryopreservation. To address this problem, we previously developed a chemical dechorionation method using potassium hydroxide (KOH) and sodium hypochlorite (NaClO), which effectively removed the chorion of silkworm embryos and enabled embryo development in a dry–moist culture system [31]. However, the development of a permeabilization method remains to be achieved.

The purpose of this study was to assess the effects of different permeabilization treatments on the permeability, development, hatching, and viability of silkworm embryos. We developed a method for the reliable permeabilization of dechorionated silkworm embryos that is easy to handle and compatible with the dry–moist culture system. The findings revealed that a specific embryonic stage maintained high permeability and viability after permeabilization treatment. The method developed here could be applicable to the development of cryopreservation methods for silkworms, as well as other insects.

## 2. Materials and Methods

### 2.1. Collection of Eggs and Embryonic Stages

Two silkworm strains, pnd-w1 and w1, were used in this study. The strain pnd-w1 is a mutant with a nondiapause nature and white-colored eggs, whereas w1 is a diapausing strain that requires acid treatment to break diapause and also has white-colored eggs. Male and female newly emerged moths were mated for at least 2 h at 25 °C and then stored for one day at 5 °C, which allowed female moths to lay easily after placing them at 25 °C. The females were separated, transferred to a piece of paper, and kept in dark places at 25 °C for one hour. The eggs laid on the papers were incubated at 25 °C until the defined time. At 20 h after egg laying (AEL), the eggs of w1 were immersed in a hydrochloric acid solution (HCl diluted with distilled water; specific gravity at 15 °C: 1.1100) at 25 °C for 90 min and then rinsed in running water for 30 min in order to break diapause. We defined the embryonic stages as described by Urbán-Duarte et al. [31]. The early embryonic stages were defined as “≤Stage 17” and the late embryonic stages as “≥Stage 19”.

### 2.2. Dechorionation and Permeabilization Processes

The eggs were dechorionated as described by Urbán-Duarte et al. [31]. Briefly, the eggs were attached to a nylon net (30 µm) and exposed to 30% KOH for 7 min and 2% NaClO (aqueous bleach, Haiter, Kao, Japan: 6% chlorine content) for 5 min at 27 °C, rinsed in phosphate-buffered saline (PBS; pH 7.4, 302 mOsm, Takara, Japan) for 10 min, immersed in 1% sodium carbonate (Na_2_CO_3_) for 1 min, and rinsed in PBS for 5 min.

**Figure 1:**
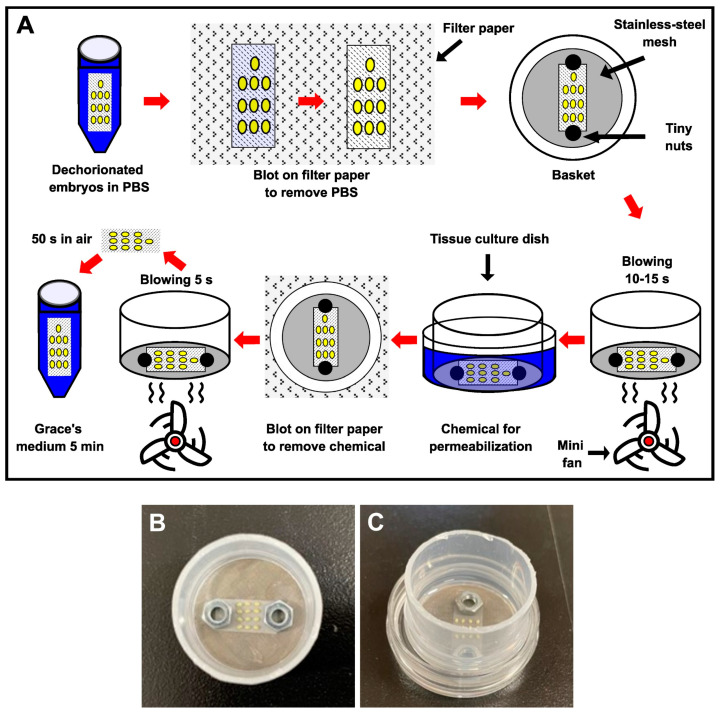
Method for permeabilization of dechorionated silkworm eggs. (**A**) Outline of the permeabilization method. A nylon net with dechorionated eggs was blotted on filter paper using tweezers, placed into a basket, and clamped with tiny nuts. The remaining PBS around the eggs was removed by blowing air with a fan. The embryos were then exposed to a chemical (hexane, heptane, triton-100, or Tween-80) for permeabilization, which was removed by blotting the basket on filter paper, followed by blowing air with a fan. The eggs were kept in room air and transferred to Grace’s insect medium. (**B**) Image showing dechorionated eggs clamped onto the bottom of the basket with tiny nuts. (**C**) Image demonstrating dechorionated eggs in the basket immersed in the tissue-culture dish with chemicals for permeabilization.

Prior to permeabilization, the nylon net with dechorionated eggs was removed from the PBS and blotted on sterilized filter paper to eliminate excess PBS (Figure 1A). The dechorionated eggs were then placed in a basket with a stainless-steel mesh (140 µm) strainer and clamped onto the bottom of the basket with tiny weights to prevent the nylon net from lifting or moving during treatment (Figure 1A,B); this allowed the eggs to be held on the nylon net, treating them more gently, and avoiding damage during the experimental treatment. The basket with dechorionated eggs was dried by blowing air using a small fan for 15 s and then exposed to chemicals, including hexane, heptane, Triton X-100, and Tween-80 (all from Wako, Japan), to permeabilize the embryos (Figure 1A,C). Each chemical was used as follows: 30 s of hexane and heptane, 2 min of 7% (*v*/*v*) Triton X-100 solution in distilled water [28], 2 min of 0.08% (*v*/*v*) Tween-80 solution in distilled water [30]. The chemicals were removed by blotting the basket on sterilized filter paper, followed by blowing air using a small fan for 5 s (Figure 1A). Following that, the eggs were kept in room air for 50 s, and the permeabilized eggs were transferred to Grace’s insect medium (Sigma-Aldrich) for 5 min (Figure 1A) and cultured using the dry–moist method [31]. All of these steps were performed in an air-conditioned room (25 °C).

### 2.3. Rhodamine B Staining

To evaluate the permeability of the chemicals in the embryos, a modified simple-staining method described by Mazur et al. [23] was used. Briefly, dechorionated eggs attached to a nylon net before and after permeabilization treatment were dyed in a solution of 0.1% (*w*/*v*) rhodamine B (Wako, Japan; molecular weight (MW) = 479.02) in PBS for 10 min, rinsed in PBS twice for 1 min, and then placed in a tissue-culture dish (40 × 13.5 mm; AS ONE Co., Nishi-ku, Japan) for visualization using a microscope. To investigate whether rhodamine B reaches the embryos through the membranes (lipid layer, vitellin, and serosal) following rhodamine B staining, naked embryos (free of membranes) derived from eggs were visualized. The eggs were transferred to a tissue culture dish (40 × 13.5 mm; AS ONE Co.) containing 0.1% (*w*/*v*) polyvinyl alcohol solution (PVA) in PBS. The PVA solution prevented the embryos from adhering to the tissue-culture dish during the process. The eggs were then dissected under a stereomicroscope using a needle and tweezers to remove the membranes. Naked embryos were rinsed twice with a PVA solution prior to visualization under a microscope. Embryos showing rhodamine B uptake (stained red) were considered permeabilized. Embryos were imaged using a stereomicroscope (SMZ 745; Nikon, Japan) with a digital camera (SIGHT DS-Vi1; Nikon, Japan).

### 2.4. Osmotic Response of Embryos to Sucrose and Ethylene Glycol (EG), and Data Analysis

The experimental design is shown in Figure S1. Dechorionated eggs attached to a nylon net with or without permeabilization treatment were washed twice in PBS. The eggs were then placed in a tissue culture dish (40 × 13.5 mm, AS ONE Co.) and clamped to the dish with tiny weights (Figure S1A,B). Following that, 2 mL of PBS was added to the eggs, they were incubated for 5 min, and the osmotic response of the eggs to sucrose (Wako, Japan) was examined. Once the PBS was removed from the eggs, 2 mL of a 1 M sucrose solution in PBS was added, and the response images of the eggs were taken using a stereomicroscope (SZX16; Olympus Co., Shinjuku City, Japan) with a digital camera, DP71, and digitalized by a time-lapse system every 5 min for 30 min using the cellSens Standard software (Olympus Co., Japan). All this process was performed in an air-conditioned room (25 °C). Experimental data were obtained by measuring the cross-sectional area of each digitalized egg on a computer using ImageJ software (NIH, Bethesda, MD, USA; https://imagej.nih.gov/ij/ accessed on 20 July 2021). The cross-sectional area digitalized was separated out around the contour of the membranes (vitellin and serosal), calculated (Figure S1C), and then the shrinkage changes against the initial area in PBS were represented as the “relative area”. Only eggs with a shape similar to that of an ellipse were investigated; eggs that had substantial morphological changes were damaged or did not show a typical shrink response were not included in the analysis. Additionally, the same experiments were performed using EG instead of sucrose.

### 2.5. EG Treatment in Viability Assay

The permeabilized embryos attached to nylon were exposed to a 2 M EG (Wako, Japan) solution in Grace’ medium in each of the exposure times (30, 60, 90, and 120 min). Next, the embryos were blotted on a sterilized filter paper to remove the excess EG solution and step-wisely transferred to Grace’s medium containing decreasing amounts of trehalose (0.5 M for 5 min; 0.25 M for 10 min; 0.125 M for 10 min; Wako, Japan) to remove the EG. Then, the embryos were blotted on a sterilized filter paper to remove the excess trehalose solution and washed in Grace’s medium two times for 5 min. All these steps were performed in an air-conditioned room (25 °C). Finally, the embryos were cultured in the dry–moist system.

### 2.6. Assessment of Development and Viability

Embryos from each experiment were examined under a stereomicroscope (LED300SLI, Leica Microsystems, Wetzlar, Germany) to assess development and viability. Embryos showing appropriate developmental progress at 216 h AEL were scored as “developing embryos”. Embryos with head formation and complete ingestion of the membranes encasing the embryos were scored as “serosa ingestion”. Larvae waking at 11 d AEL were scored as “hatching”. Larvae after the first molting on day 19 AEL were scored as “second instar”. The number of embryos developing into “moths” was recorded. The female and male moths were mated for at least 2 h with untreated moths of the same strain. After mating, the females were separated, transferred to a piece of paper, and kept in dark places at 25 °C for 1 d to collect eggs. After 20 h AEL, eggs from the w1 strain, although not pnd-w1, were treated with acid to break diapause. The eggs were incubated at 25 °C for 12 d and the hatchability was examined. The moths with progeny were defined as “fertile moths”. The percentage of serosa ingestion, hatching, and second instar were calculated to the total number of embryos used for each experimental replicate.

### 2.7. Statistical Analysis

SPSS software (version 27.0; Armonk, NY, USA: IBM Co.) was used for all analyses. Development and viability data were analyzed using the Kruskal–Wallis test and post hoc Dunn’s test to compare each experimental group, while the Mann–Whitney test was used to compare permeabilized pnd-w1 and w1 embryos. Osmotic-response data were examined using analysis of variance (ANOVA) and Tukey’s post hoc test to compare each experimental group. Statistical significance was set at *p* < 0.05. The replicates for each experiment were performed using eggs from the same pool of female moths laid which hatched within 5 days.

## 3. Results

### 3.1. Improvements in the Permeabilization Process

In a previous study, a dechorionation method for the eggs was developed, where eggs attached to a nylon net were treated with KOH and NaClO to make handling easy and to reduce handling damage during the dechorionation treatment [31]. However, since permeabilization treatments to remove the lipid layer require other solutions, such as hexane, which could detach eggs from the nylon net, we developed a system to treat eggs more gently with these solutions (Figure 1). As a result, this system prevented the detachment of the eggs from the nylon net, which allowed control of the exposure time, reduced the annoyance of handling work, and reduced damage from the treatments.

### 3.2. Permeability at Different Embryonic Stages

**Figure 2:**
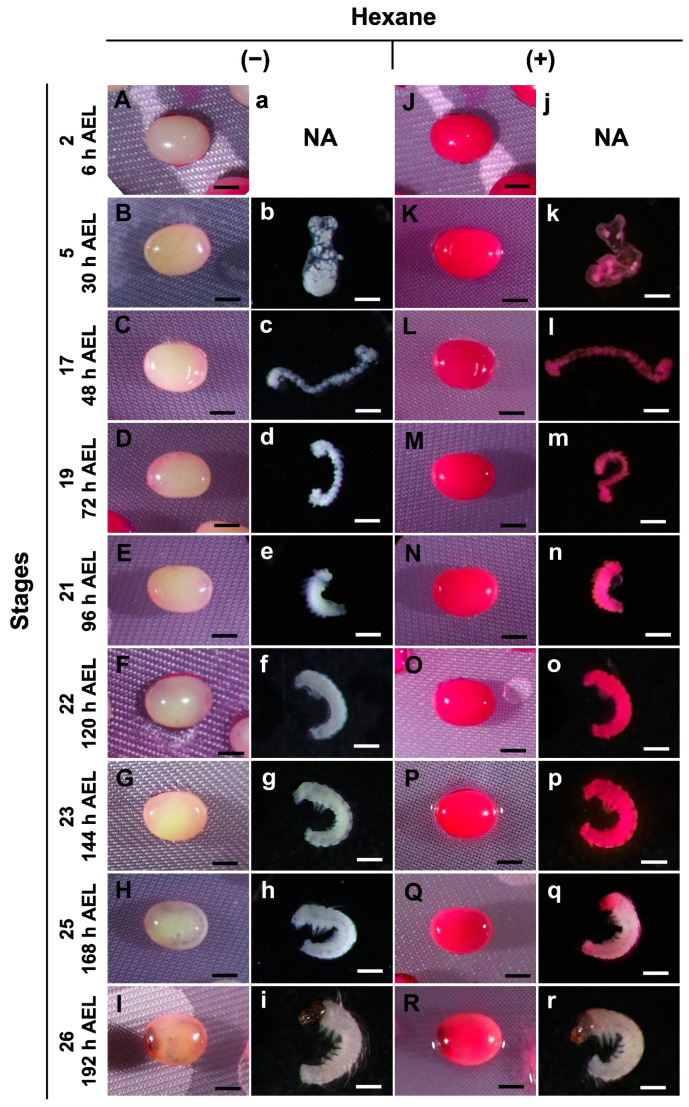
Images of permeabilized embryos at different stages of the pnd-w1 strain. The dechorionated eggs with (+) or without (−) permeabilization treatment with hexane for 30 s were treated with 0.1% rhodamine B for 10 min. (**A**–**I**) Nonpermeabilized eggs treated with hexane. (**a**–**i**) Naked embryos derived from (**B**–**I**) eggs. (**J**–**R**) Permeabilized eggs treated with hexane. (**j**–**r**) Naked embryos derived from (**K**–**R**) eggs. Permeabilized embryos (stained red). Stage 2, cleavage; stage 5, germband formation 2; stage 17, the appearance of abdominal appendages; stage 19, shortening stage; stage 21, blastokinesis; stage 22, completion of blastokinesis; stage 23, the appearance of trichogen cells; stage 25, the appearance of taenidium; stage 26, head pigmentation I; NA, not available. Scale bar: 500 µm.

The effects of permeabilization at different embryonic stages were investigated using hexane and the pnd-w1 strain, which does not require acid treatment to break the diapause nature of embryos. For this purpose, a method using a 0.1% rhodamine B solution [23] was very useful. Dechorionated eggs at stages ranging from stage 2 (cleavage, 6 h AEL) to stage 26 (head pigmentation one, 192 h AEL) were treated with hexane for 30 s for permeabilization, immersed in a 0.1% rhodamine B solution for 10 min, and then evaluated. Unpermeabilized eggs were used as controls. The eggs without permeabilization were not stained by rhodamine B at any of the stages (Figure 2A–I); similarly, the naked embryos were free of membranes, such as the lipid layer, vitelline, and serosal (Figure 2b–i). In contrast, the permeabilized eggs were stained with rhodamine B at all stages (Figure 2J–R). The naked embryos from stage 2 to stage 23 performed similarly, though not for stages 25 and 26 (Figure 2k–r). These results indicate that hexane treatment was significantly effective in improving the permeability of the silkworm embryos, whereas embryos in the later stages did not take up rhodamine B, even if permeabilization was performed.

**Figure 3:**
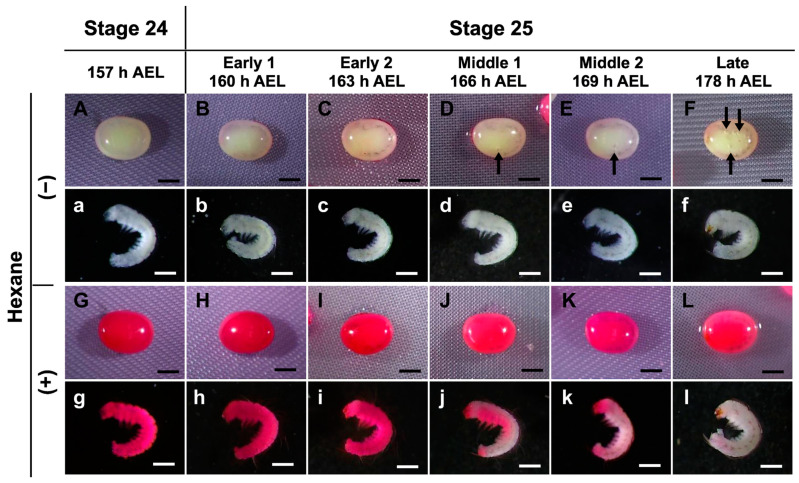
Permeability of embryos during stages 24 and 25 (appearance of taenidium) of the pnd-w1 strain. The dechorionated eggs were subjected to a permeabilization treatment with hexane for 30 s, immersed in 0.1% rhodamine B for 10 min, and then rinsed with PBS. (**A**–**F**) Eggs without hexane treatment. (**a**–**f**) Naked embryos derived from (**A**–**F**) eggs. (**G**–**L**) Eggs with hexane treatment. (**g**–**l**) Naked embryos from (**G**–**L**) eggs. Black arrows indicate air bubbles at the start of respiration. Permeabilized embryos are those stained red. Scale bar: 500 µm.

The uptake of rhodamine B was prevented in late embryos at stages 25 and 26 (Figure 2); therefore, we investigated the stage boundary in detail for staining with rhodamine B. In the first step, we used a stereomicroscope to examine the eggs from 157 to 178 h AEL (stages 24 and 25) every three hours to determine the differences in embryonic development. As shown in Figure 3, the trachea was observed after 160 h AEL (early 1 stage 25) though not before 157 h AEL (stage 24). Furthermore, after 166 h AEL (middle 1 stage 25), air bubbles were observed inside the eggs, and the mouth parts were black at 178 h AEL (late stage 25). After permeabilizing the eggs and staining with rhodamine B, the staining of the non-naked embryos was intense until 163 h AEL (early 1 stage 25); however, embryos from 166 h AEL were less stained. Similarly, the results for the naked embryos were clearer. This result indicated that there was an apparent difference in the permeability of rhodamine B between 163 h and 166 h AEL.

### 3.3. Osmotic Response of Embryos to Sucrose

**Figure 4:**
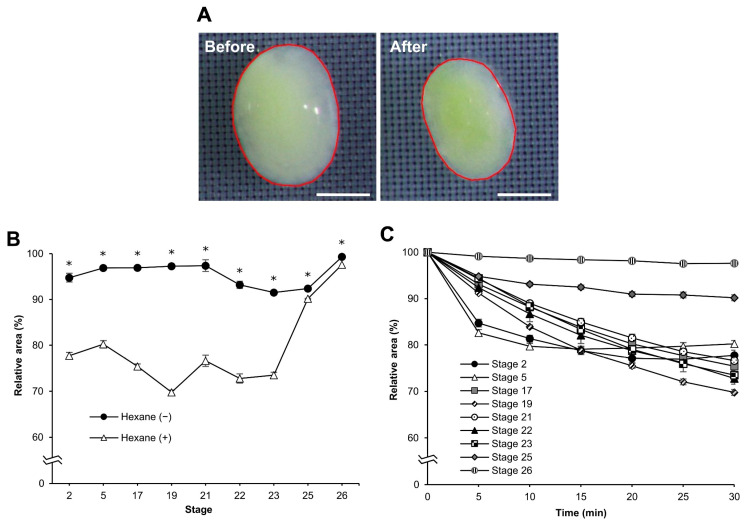
The cross-sectional changes in eggs at different embryonic stages of the pnd-w1 strain. The dechorionated eggs without and with permeabilization treatment of hexane for 30 s were exposed to 1 M sucrose solution for 30 min at 25 °C. (**A**) Eggs in the embryonic stage 23 before and after exposure to sucrose solution for 30 min. The red lines indicate the separated area as the cross-sectional area of eggs digitalized on a computer. Scale bar: 500 µm. (**B**) The relative area changes in each different stage. Hexane (−) and (+) represent with and without permeabilization treatment with hexane, respectively. (**C**) The relative area changes of permeabilized eggs in each embryonic stage through the exposure time to sucrose solution. The experiment was replicated four times (n = 4); each replicate consisted of 8–12 eggs. Error bars represent the standard error (SE), and * represents a significant difference at 30 min, *p* < 0.05. ANOVA was used for statistical analysis.

Water fluidity changes in dechorionated eggs after permeabilization. We examined the osmotic response of the eggs to sucrose. When the eggs were exposed to a hypertonic solution of 1 M sucrose, they shrank, as shown by changes in their cross-sectional areas (Figure 4A). We measured the changes in the cross-sectional area of the dechorionated eggs to sucrose following permeabilization treatment at each different stage as the “relative area”. There were obvious differences between eggs with and without hexane treatment at embryonic stages 2–23, though less so at stages 25 and 26 (Figure 4B). Their significant differences were as follows: stage 2, *F* = 199.776, *p* = 0.000; stage 5, *F* = 197.025, *p* = 0.000; stage 17, *F* = 566.257, *p* = 0.000; stage 19, *F* = 1761.489, *p* = 0.000; stage 21, *F* = 980.390, *p* = 0.000; stage 22, *F* = 130.895, *p* = 0.000; stage 23, *F* = 201.500, *p* = 0.000; stage 25, *F* = 8.849, *p* = 0.024; stage 26, *F* = 272.510, *p* = 0.000. When the relative areas during sucrose treatment were compared at each stage, those of the earlier stages (stages 2–23) decreased faster than those of the later stages (stages 25 and 26). However, the relative areas of stages two and five rapidly decreased compared to the other early stages in the early time (up to 5 min) of sucrose treatment and then appeared to reach a plateau at 15 min (Figure 4C).

### 3.4. More Detailed Analysis of the Osmotic Response during Embryonic Developmental Stages

**Figure 5:**
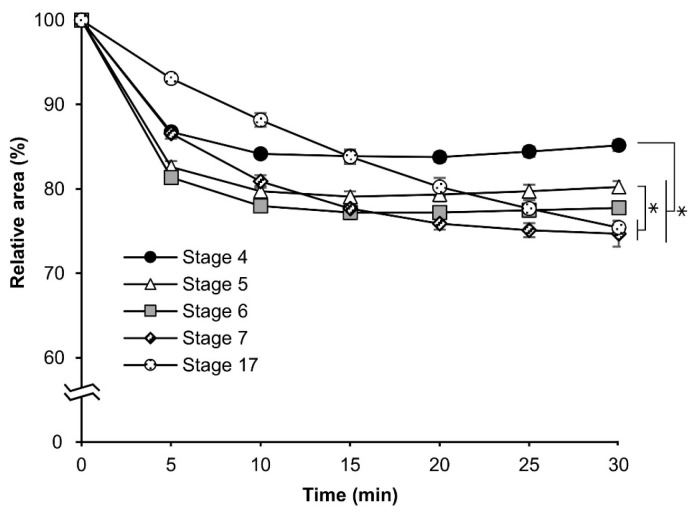
The cross-sectional changes of eggs at early embryonic stages of the pnd-w1 strain during exposure to a sucrose solution. The dechorionated eggs were permeabilized with hexane for 30 s and exposed to a 1 M sucrose solution for 30 min at 25 °C. The experiment was replicated four times (n = 4); each replicate consisted of 8–12 eggs. Error bars represent the SE, and * represents differences at 30 min, *p* < 0.05. ANOVA and post hoc Tukey’s test were used for the statistical analysis.

Different osmotic responses were observed in eggs at each embryonic stage. As a result, we evaluated the permeability of eggs from embryonic stage 4 (germband formation one, 24 h AEL) to stage 17 (appearance of abdominal appendages, 48 h AEL) after permeabilization treatment. The experiment was performed as mentioned in Section 3.3. As shown in Figure 5, the relative-area rates decreased depending on the earliness of the stage, although with the exclusion of stages 7 (germband formation four, 42 h AEL) and 17. This finding indicated that a change in the quality of the contents inside the egg might have occurred. The response curves of stages 7 and 17 were slower than those of the other stages: stage four, stage five (germband formation two, 30 h AEL), and stage six (germband formation three, 36 h). The relative areas at 30 min were 85.2%, 80.2%, 77.8%, 74.7%, and 75.4% for stages 4, 5, 6, 7, and 17, respectively.

The significant differences were as follows: stage 4 vs. stage 5, *p* = 0.002; stage 4 vs. stage 6, stage 7, stage 17, *p* = 0.000; stage 5 vs. stage 7, *p* = 0.001; and stage 5 vs. stage 17, *p* = 0.002.

**Figure 6:**
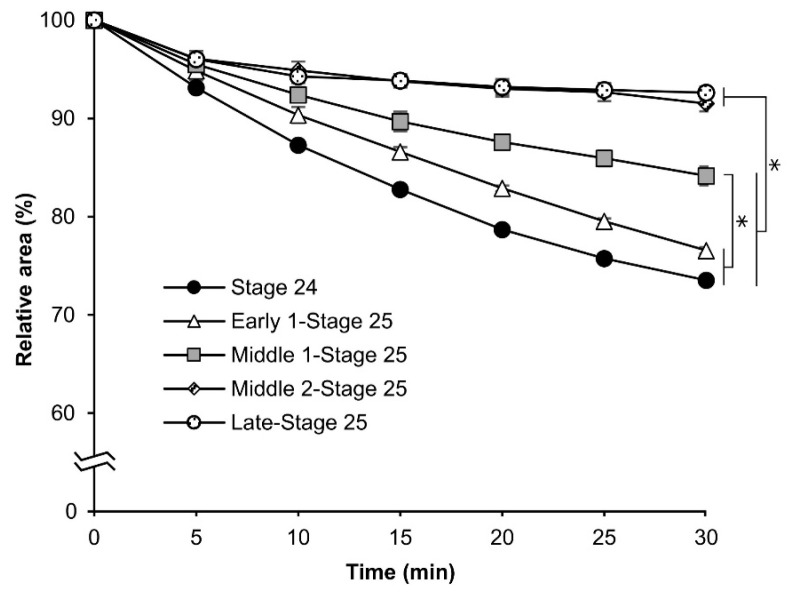
The cross-sectional changes of eggs at embryonic stage 24 (appearance of setae) and different phases of stage 25 (appearance of taenidium) during exposure to a 1 M sucrose solution. The dechorionated eggs of the pnd-w1 strain were permeabilized with hexane for 30 s and exposed to a sucrose solution for 30 min at 25 °C. The experiment was replicated three times (n = 3); each replicate consisted of 10–12 eggs. Error bars represent the SE, and * represents differences at 30 min, *p* < 0.05. ANOVA and post hoc Tukey’s test were used.

Based on the rhodamine B uptake experiment shown in Figure 3, we evaluated the osmotic response of eggs at embryonic stage 24, early 1 stage 25, middle 1 stage 25, middle 2 stage 25, and late stage 25. As shown in Figure 6, the relative area was reduced with embryonic development. The relative areas of the eggs at embryonic stage 24 and early 1 stage 25 decreased significantly faster than those at middle 1 stage 25, middle 2 stage 25, and late stage 25. Each significant difference was as follows: middle 1 stage 25 vs. stage 24 and early 1 stage 25, *p* = 0.000; middle 2 stage 25, and late stage 25 vs. all others, *p* = 0.000.

### 3.5. Viability of Embryos after Permeabilization

**Figure 7:**
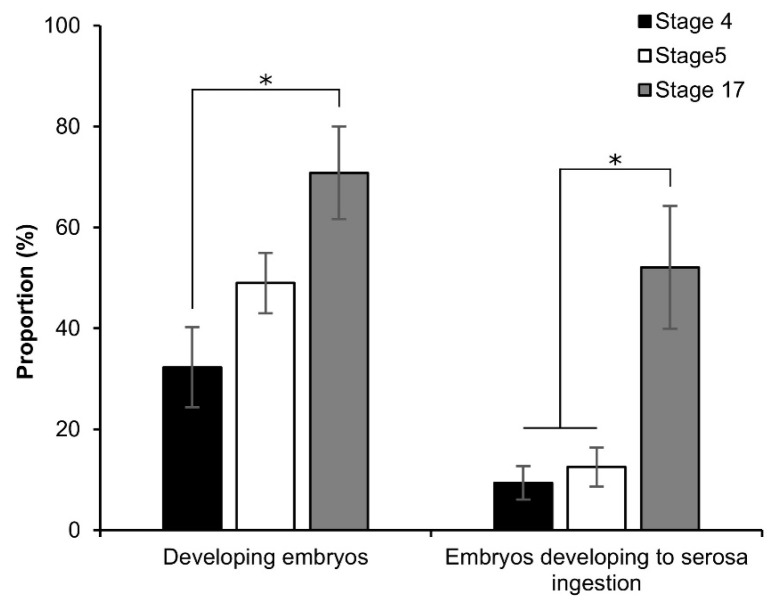
Effect of permeabilization treatment on the viability of early embryonic stages. The dechorionated eggs of the pnd-w1 strain were permeabilized with hexane for 30 s. Stage 4, germband formation 2, 24 h AEL; stage 5, germband formation 2, 30 h AEL; stage 17, the appearance of abdominal appendages, 48 h AEL. The experiment was replicated eleven times (n = 11); each replicate consisted of 12 embryos. Error bars represent the SE. * *p* < 0.05. Kruskal–Wallis test and post hoc Dunn’s test were used for the statistical analysis.

To investigate the viability of the embryos, the early embryonic stages after permeabilization were first cultured using the dry–moist method [31]. However, after 1 d of incubation, the permeabilized eggs formed an abnormal shape (Figure S2A), and the next day, the eggs burst. As a result, we improved a new culture method that was modified from the culture system using liquid paraffin developed by Okada [32]. Briefly, after permeabilization treatment, the nylon net with eggs was placed on a tissue-culture dish (40 × 13.5 mm, AS ONE Co.) and clamped to the dish with sterilized weight (Figure S2B); 2 mL of liquid paraffin (Wako, Japan) was added to the dish, and it was then incubated at 25 °C for 9 d. Owing to the improved culture system, the embryos developed to serosa ingestion (Figure 7 and Figure S2C,D). The proportion of developing embryos increased depending on embryonic growth: 32.3%, 49.0%, and 70.8% at stages 4, 5, and 17, respectively; the proportion of stage 4 was significantly lower than that of stage 17 (*z* = 2.847; *p* = 0.004). While stages 4 and 5 were significantly lower than stage 17 in the proportion of embryos that developed to serosa ingestion: stage 4 vs. stage 17, *z* = 2.483, *p* = 0.013; stage 5 vs. stage 17, *z* = 2.051, *p* = 0.040.

**Figure 8:**
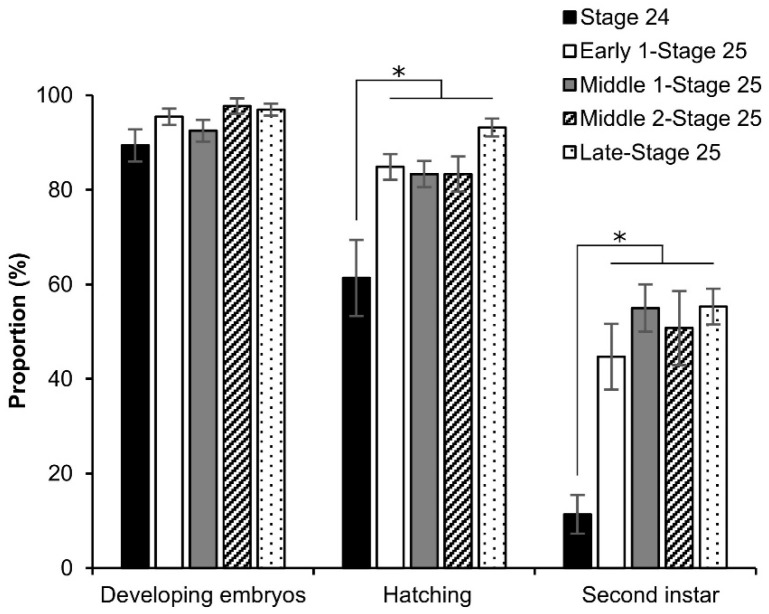
Effect of permeabilization treatment on the viability of late embryonic stages. The dechorionated eggs of the pnd-w1 strain were permeabilized with hexane for 30 s. The experiment was replicated eleven times (n = 11); each replicate consisted of 12 embryos. Error bars represent the SE. * *p* < 0.05. Kruskal–Wallis test and the post hoc Dunn’s test were used for the statistical analysis.

Furthermore, we investigated the effects of permeabilization treatment on the viability of the late stage (around stage 25) using the dry–moist culture method [31]. As shown in Figure 8, all phases of stage 25 resulted in considerable growth up to the second instar, though not stage 24; however, the phases of stage 25 showed a slight decrease in the embryos that developed to the second instar. There was a significant difference in stage 24 against the other phases: hatching (stage 24 vs. early 1 stage 25, *z* = 2.878, *p* = 0.003; stage 24 vs. middle 1 stage 25, *z* = 3.843, *p* = 0.000; stage 24 vs. middle 2 stage 25, *z* = 3.588, *p* = 0.000; stage 24 vs. late stage 25, *z* = 4.018, *p* = 0.000), and second instar (stage 24 vs. early 1 stage 25, *z* = 1.918, *p* = 0.055; stage 24 vs. middle 1 stage 25, *z* = 1.918, *p* = 0.055; stage 24 vs. middle 2 stage 25, *z* = 1.918, *p* = 0.055; stage 24 vs. late stage 25, *z* = 1.918, *p* = 0.055).

### 3.6. Effect of Hexane Exposure Time

**Figure 9:**
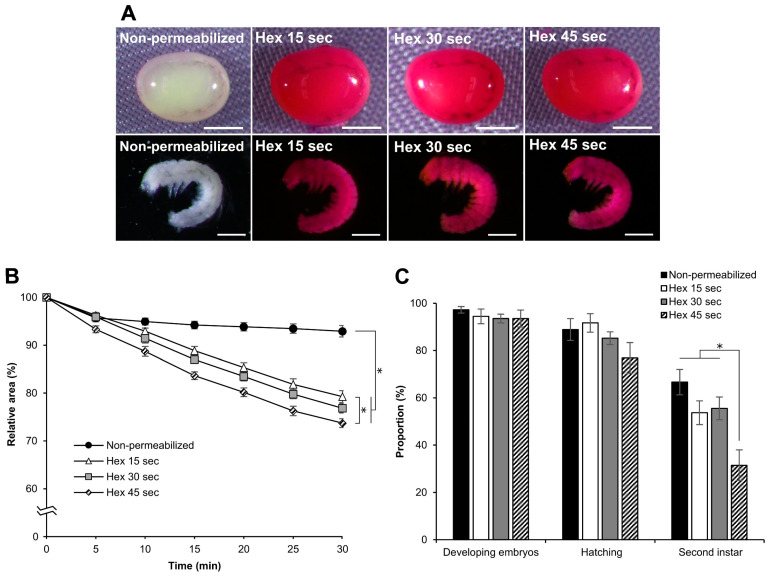
Effect of permeabilization treatment at different hexane exposure times. The dechorionated eggs of the pnd-w1 strain at early 1 stage 25 were permeabilized with hexane for 15, 30, and 45 s. (**A**) Images of embryos dyed in 0.1% rhodamine B for 10 min. Scale bar: 500 µm. (**B**) The dynamic area changes of eggs during exposure to a 1 M sucrose solution for 30 min at 25 °C. The experiment was replicated five times (n = 5); each replicate consisted of 8–12 eggs; * represents differences at 30 min, *p* < 0.05. ANOVA and post hoc Tukey’s test were used for the statistical analysis. (**C**) The proportion of the developing embryos, the hatching embryos, and the embryos developed to the second instar larvae in each examined treatment. The experiment was replicated nine times (n = 9); each replicate consisted of 12 embryos; * *p* < 0.05. Kruskal–Wallis test and the post hoc Dunn’s test were used for the statistical analysis. Error bars represent the SE.

To determine the optimal conditions for permeabilization using hexane, we treated dechorionated eggs with different exposure times. We performed permeabilization treatments with hexane for 15, 30, or 45 s to examine rhodamine B uptake, response to sucrose, and viability of the embryos. All eggs treated at each time point were stained with rhodamine B, along with all the naked embryos (Figure 9A). In the embryonic response to sucrose, there were differences in the decrease in the relative area (Figure 9B). The significant differences at 30 min were as follows: hex 45 s vs. hex 15 s: *p* = 0.019, nonpermeabilized vs. hex 15 s, *p* = 0.000; nonpermeabilized vs. hex 30 s, *p* = 0.000; nonpermeabilized vs. hex 45 s, *p* = 0.000. The effects of different hexane exposure times on embryo viability were not observed during embryonic development or hatching. However, significant differences were observed in the proportion of embryos developing to the second instar larvae (Figure 9C). These were as follows: hex 45 s vs. hex 15 s: *z* = 1.968, *p =* 0.048; hex 45 s vs. hex 30 s, *z* = 2.183, *p =* 0.028; hex 45 s vs. nonpermeabilized, *z* = 3.451, *p =* 0.000). Consequently, we concluded that treatment for 30 s was the best for permeabilization, maintaining considerable viability.

### 3.7. Effect of Permeabilization with Chemicals Other Than Hexane

**Figure 10:**
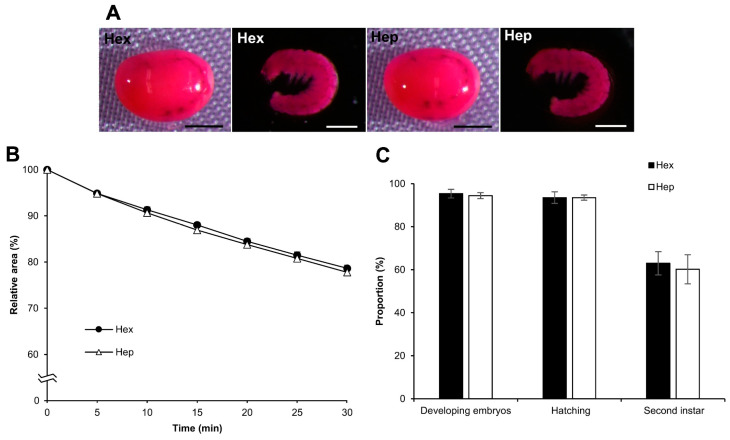
Effect of permeabilization treatment with hexane and heptane. The dechorionated eggs at the embryonic early 1 stage 25 of the pnd-w1 strain were permeabilized with hexane (hex) or heptane (hep) for 30 s. (**A**) The images of embryos treated with 0.1% rhodamine B for 10 min. Scale bar: 500 µm. (**B**) The relative areas of eggs exposed to a 1 M sucrose solution for 30 min at 25 °C. The experiment was replicated five times (n = 5); each replicate consisted of 10–12 eggs. (**C**) The proportions of the developing embryos, the hatching embryos, and the embryos developed to the second instar larvae in each treatment. The experiment was replicated five times (n = 9); each replicate consisted of 12 embryos. Error bars represent the SE.

**Figure 11:**
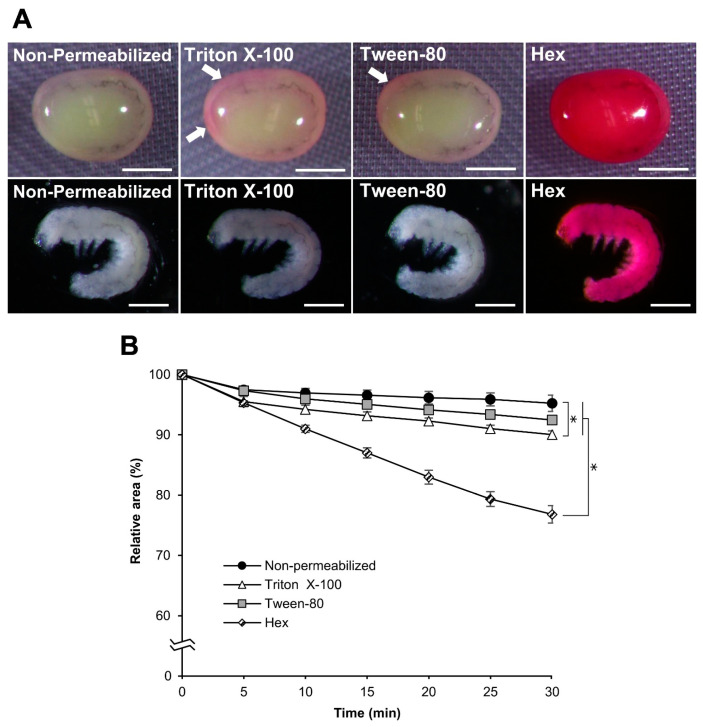
Effect of permeabilization treatment with Triton X-100 and Tween-80. Each dechorionated egg at the embryonic early 1 stage 25 of the pnd-w1 strain was permeabilized with 7% Triton X-100 for 2 min, 0.08% Tween-80 for 2 min, or hexane (hex) for 30 s. (**A**) The images of embryos treated with 0.1% rhodamine B for 10 min. Scale bar: 500 µm. The white arrows indicate a small uptake of rhodamine B. Scale bar: 500 µm. (**B**) The relative areas of eggs exposed to a 1 M sucrose solution for 30 min at 25 °C. The experiment was replicated four times (n = 4); each replicate consisted of 10–12 eggs. Error bars represent the SE. * represents the differences at 30 min, *p* < 0.05. ANOVA and the post hoc Tukey’s test were used for statistical analysis.

We examined the influence of chemicals other than hexane, including alkane (heptane) and surfactants (Triton X-100 and Tween-80), on embryo permeabilization. Experiments with these chemicals were performed in the same manner as those with hexane. Using heptane, the results were almost the same as those obtained for hexane in each experiment for rhodamine B uptake, the osmotic response to sucrose, and embryonic viability. No significant differences were observed between hexane and heptane (Figure 10), while the effects of Triton X-100 and Tween-80 on permeabilization were completely different from those of hexane. As shown in Figure 11A, rhodamine B uptake in the eggs after treatment with Triton X-100 and Tween-80 was visibly lower than that of hexane, even though a small rhodamine uptake was observed for Triton X-100 and Tween-80. Similarly, the responses of Triton X-100 and Tween-80 to the sucrose solution were different, where the shrinkage rates were significantly lower than those of hexane: hex vs. Tween-80, *p* = 0.000; hex vs. Triton X-100, *p* = 0.000 (Figure 11B), and non-permeabilization vs. Triton X-100, *p* = 0.010.

### 3.8. Effect on Different Strains: Diapausing

**Figure 12:**
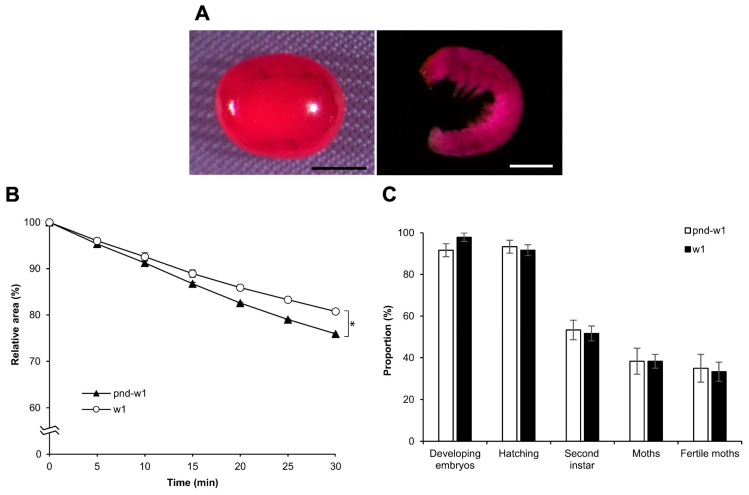
Effect of permeabilization treatment on eggs of the diapause strain. The dechorionated eggs of a diapause strain, w1, were permeabilized with hexane for 30 s. (**A**) The images of an egg and an embryo treated with 0.1% rhodamine B for 10 min. Scale bar: 500 µm. (**B**) The relative areas of embryos exposed to 1 M sucrose solution for 30 min at 25 °C. The experiment was replicated five times (n = 5); each replicate consisted of 10–12 eggs. An ANOVA test was used for statistical analysis. (**C**) The proportions of the developing embryos, the hatching embryos, and the embryos developed to the second instar larvae in each treatment. The experiment was replicated five times (n = 5); each replicate consisted of 12 embryos. The Mann–Whitney test was used for the statistical analysis. Error bars represent the SE. * represents the differences at 30 min, *p* < 0.05.

Overall, the findings indicated that permeabilization treatment with hexane for 30 s was most effective for the embryos of the silkworm, *B. mori*, at early 1 stage 25. To examine whether the method could be valid for a different strain, we performed the same experiments using w1 eggs with a diapause nature: rhodamine B uptake, the response to sucrose, and the viability of embryos. Consequently, there was no difference in rhodamine B uptake and embryo viability between the pnd-w1 and w1 strains; however, there was a significant difference in the response to sucrose *(F* = 49.320, *p* = 0.000; Figure 12B). Notably, both strains were fertile in the next generation (Figure 12C; Table S1).

### 3.9. Osmotic Response of Embryos to EG and Viability

**Figure 13:**
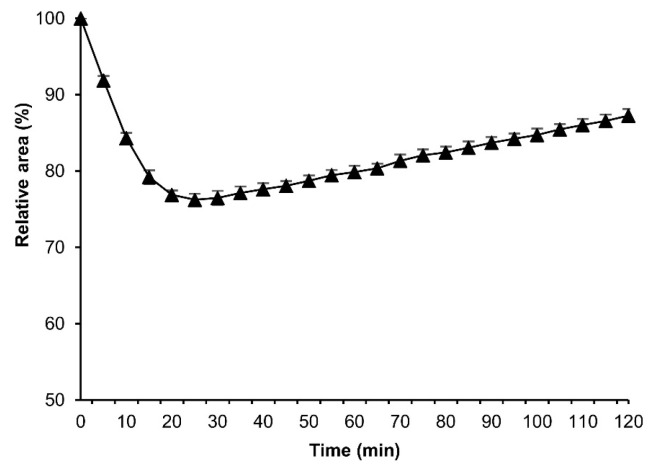
The cross-sectional changes of the eggs at embryonic early stage 25 during exposure to EG solution. The dechorionated eggs of the pnd-w1 strain were permeabilized with hexane for 30 s and exposed to 2 M EG solution for 120 min at 25 °C. The experiment was replicated three times (n = 3); each replicate consisted of 10–12 eggs. Error bars represent the SE.

To assess the permeability of a cryoprotective agent (CPA) into the embryos, we performed the osmotic-response experiments using EG instead of sucrose. The permeabilized eggs of the embryonic early stage 25 were exposed to 2 M EG solution in Grace’s insect medium at 25 °C for 120 min, and then analyzed as mentioned in Section 2.4. The eggs initially shrank and gradually re-expanded; however, the re-expansions were not recovered to the initial relative area of the embryos prior to the treatment (Figure 13).

**Figure 14:**
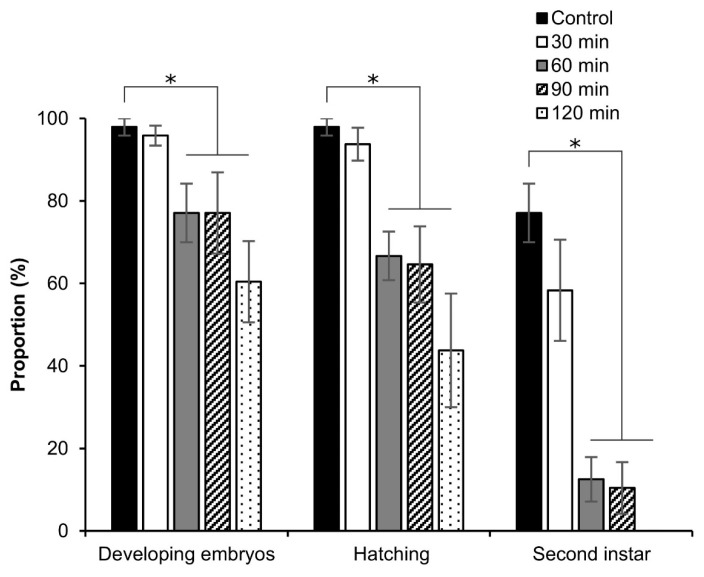
Effect of EG on the viability of silkworm embryos. The permeabilized embryos of the pnd-w1 strain at early stage 25 were exposed to 2 M EG solution for 30, 60, 90, and 120 min at 25 °C. Control represents no exposure to EG. The experiment was replicated four times (n = 4); each replicate consisted of 12 embryos. Error bars represent the SE. * *p* < 0.05. Kruskal–Wallis test and post hoc Dunn’s test were used for the statistical analysis.

Next, we investigated the tolerance of permeabilized embryos in early stage 25 to EG. As shown in Figure 14, no significant differences were found at 30 min of exposure to 2 M EG with respect to the proportions of developing embryos, rates of hatching, and embryos developed to the second instar, compared to embryos not exposed to EG. By contrast, at 60, 90, and 120 min of exposure to 2 M EG, a significant decrease in the proportions of developing embryos (60 min: z = 2.145, *p* = 0.031; 90 min: z = 2.022, *p* = 0.043; 120 min: z = 3.003, *p* = 0.002) and rates of hatching (60 min: z = 2.090, *p* = 0.036; 90 min: z = 2.272, *p* = 0.023; 120 min: z = 3.120, *p* = 0.001) was found. Moreover, a dramatic decrease in the proportions of embryos developed to the second instar was observed (60 min: z = 2.234, *p* = 0.025; 90 min: z = 2.479, *p* = 0.013); no embryos developed to the second instar at 120 min (Figure 14).

## 4. Discussion

The lack of embryonic permeability of water and other molecules could be attributed to the lipid layer on the surface of the vitelline membrane, which plays a key role in desiccation resistance [12,33]. A method for the permeabilization of embryos using alkanes, such as hexane and heptane, has been developed for dipteran species [23,25,26]. Permeabilization with hexane treatment has also been reported in lepidopterans, including *B. mori* (Lepidoptera: Bombycidae) [34] and *Pectinophora gossypiella* (Lepidoptera: Gelechiidae) [28]. However, permeabilization studies on the silkworm *B. mori* remain inadequate due to the difficulty of removing the chorion (dechorionation). In a previous study, we successfully dechorionated silkworm eggs by chemical treatment combined with a dry–moist culture, which enabled the development of embryos [31], making research on permeabilization in silkworms possible.

In this study, we developed an easy-to-handle method for the permeabilization of silkworm embryos, which enabled the assessment of permeability and viability (Figure 1). Permeabilization treatment with hexane effectively rendered the extraembryonic membranes (vitellin and serosal) permeable to rhodamine B at all embryonic stages (Figure 2J–R). Notably, embryos free of membranes took up rhodamine B in stages 5–23 (Figure 2k–P); however, embryos at stages 25 and 26 had decreased uptake (Figure 2q,r). Similar results were observed for the osmotic response to the sucrose solution, where the degree and speed of egg shrinkage decreased in the embryos at stages 25 and 26 (Figure 4B). This decrease in water and rhodamine B permeation may be attributed to the chitinization of embryos during late embryonic stages, which has been reported in *Drosophila melanogaster* (Diptera: Drosophilidae) embryos [24]. Additionally, although no visible differences in rhodamine uptake were observed between early embryonic stages (stages 4–17; Figure S3), the shrinkage of eggs at embryonic stages 2–7 quickly proceeded in the first minutes of the exposure to the hypertonic solution but became dull after 10 min and then the shrinkage level finally reached a low level (Figure 4C and Figure 5). This phenomenon may be attributed to changes in the yolk. However, the shrinkage of eggs slowed from embryonic stage seven compared to the previous stages. This may be attributed to changes in the extraembryonic membranes since cuticle deposition on the vitelline membrane begins to occur at around 24 h AEL [35] and could progress and be completed at around 48 h AEL (Figure 4C and Figure 5). Nevertheless, the viability of embryos at the early embryonic stages was very low (Figure 7). We characterized stage 25 into four phases according to the permeability and appearance of the taenidium (Figure 3). The results of the rhodamine B uptake experiment revealed that the permeability of the embryos decreased after the early 1 stage 25. The osmotic response of embryos to the sucrose solution demonstrated similar results (Figure 6). These findings imply that there was an essential change between the early 1 stage 25 and middle 1 stage 25, which may be attributed to chitinization. Stage 25 embryos developed normally after permeabilization with hexane, though stage 24 embryos did not (Figure 8). Similarly, lower viability was reported in earlier embryos of *Anastrepha ludens* (Diptera: Tephritidae) [25]. As a result, we considered that the permeabilization of the silkworm embryo using hexane might be optimal in the early 1 stage 25. Under optimal conditions, more than 62% of the embryos of the pnd-w1 strain permeabilized at the early 1 stage 25 phase and developed into second instar larvae, which could grow into fertile moths.

The balance between permeability and viability is assumed to depend on the species [23,25,27,36]. Lepidopteran membranes are generally thicker than those of dipteran species [16,35]. Our study showed that longer exposure to hexane increased the permeability of the embryos; however, viability decreased (Figure 9). Even though the permeabilization treatment with hexane for 30 s was optimal from the point of view of rhodamine permeation, the embryonic viability was relatively lower than that of the control. These findings indicate that there are some influences of the toxic effect of hexane on embryos, in addition to osmotic shock and dehydration or overhydration during culture due to the removal of the lipid layer. Other alkanes, such as heptane and octane, have been used for the permeabilization of lepidopteran [9] and dipteran species [26,36], and their effectiveness was species dependent. Additionally, it has been reported that the hatchability of permeabilized embryos of *Lucilia sericata* (Diptera: Calliphoridae) increased when larger molecules of alkanes were used [36]. However, our results using hexane (MW = 86.18) or heptane (MW = 100.21) for 30 s indicated similar permeability and rates of embryonic viability (Figure 10). Other studies on lepidopterans have used Tween-80 and Triton-X100 to remove the lipid layer from the vitelline membrane [28,30]. We also performed permeabilization treatments with Tween-80 and Triton X-100, which resulted in slight rhodamine B uptake and shrinkage of the embryos (Figure 11), suggesting that the permeation characteristics of these surfactants may be species specific. However, their use remains to be assessed under different conditions, such as varying concentrations and exposure times, since we used the reported concentration and time as follows: 0.08% Tween-80 for 2 min [30] and 7% Triton X-100 for 2 min [28].

The success of insect embryo cryopreservation relies on the sufficient permeability of CPAs [33]. Our results showed that after exposing the embryos to EG solution, the embryos initially shrank due to water loss and gradually re-expanded as the EG permeated the membranes and embryos. In the reports in *D. melanogaster*, the embryos at late stages shrank and recovered their original shape within 20 to 25 min after exposure to EG [24,27], which indicates that the CPA has been fully loaded. In mammal embryos, the highest degree of shrinkage has been reported after seconds of exposure to CPAs [37,38,39]. In comparison to their results, the membranes of silkworm embryos seem to have a much lower permeability to water and EG than embryos of mammals and dipteran species, so we have to research more factors such as kinds and concentrations of CPAs, exposure time, embryonic stage, and silkworm strains.

The toxicity of EG to the embryos was evaluated. The exposure to EG for 30 min (Figure 14) showed normal development and similar results in favorable viability to the control; however, longer exposure times reduced the embryonic viability.

Differences exist in chorion thickness, oxygen uptake, and water loss between diapause and nondiapause strains of silkworms [35,40]. We developed an optimized permeabilization method using the nondiapause strain pnd-w1: dechorionated embryos of early 1 stage 25, hexane 30 s. We examined whether the permeabilization method optimized using the nondiapause strain was suitable for the diapause strain w1. Consequently, similar data were found in the rhodamine B uptake and viability experiments in the diapause strain, excluding the response of embryos to sucrose solutions, which showed a significant difference (Figure 12).

Ultimately, the method developed in this study allowed us to easily, effectively, and reproducibly permeabilize dechorionated silkworm eggs. This permeabilization method enabled the assessment of permeability and embryonic viability and was compatible with the previously developed dechorionation and dry-moist culture systems [31]. Our developed methods are useful for assessing the effective permeabilization of silkworm embryos in combination with the rhodamine B uptake method [13,23,27,33] and the hypertonic solution response method of embryonic shrinkage and re-expansion [27,28]. Additionally, we measured the degree of shrinkage and re-expansion using digital tools that have been used for numerous cells and embryos, including insect embryos [41], fish oocytes [42], mouse oocytes and embryos [39], and human cells and oocytes [43,44], to successfully compare each embryo under different conditions.

## 5. Conclusions

We developed a simple method for the permeabilization of dechorionated silkworm embryos, which enabled the assessment of their permeability and viability. Factors, including chemicals, exposure time, and embryonic developmental status, are critical for permeabilization and embryo viability. The permeabilization method we developed could be valuable for the study of chemicals for permeabilization and the development of cryopreservation protocols for silkworm embryos.

## Data Availability

Not applicable.

## Supplementary Materials

The following supporting information can be downloaded at: https://www.mdpi.com/article/10.3390/bioengineering10050563/s1. Figure S1: Procedure for data collection and analysis of the osmotic response of silkworm eggs; Figure S2: Images of permeabilized early embryonic stages of the pnd-w1 strain with a modified culture method using liquid paraffin; Figure S3: Images of permeabilized embryos in the early stages of the pnd-w1 strain. Table S1: Egg hatchability of fertile moths derived from permeabilized embryos of pnd-w1 and w1 strains.
