# Supplementary material for: An Effective Chemical Permeabilization of Silkworm Embryos
Source: Bioengineering (Basel). 2023 May 8;10(5):563. doi: 10.3390/bioengineering10050563 (PMC10215591; doi:10.3390/bioengineering10050563)
Supplement: Supplementary file 1 [file bioengineering-10-00563-s001.zip › bioengineering-2347405-SI.pdf]

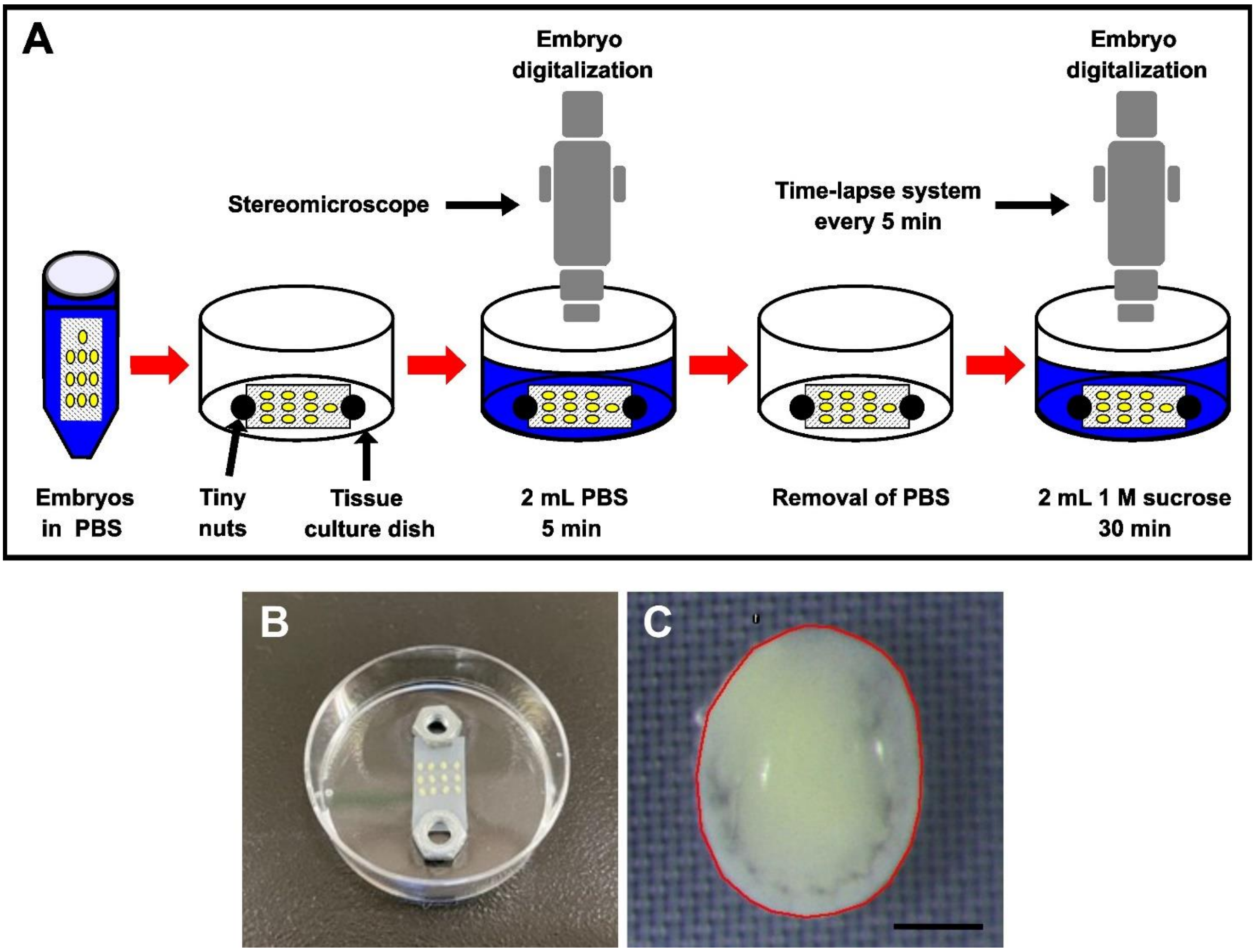


**Figure S1.** Procedure for data collection and analysis of the osmotic response of silkworm eggs. (A) A Schematic presentation of the procedure for imaging area changes in eggs during exposure to 1 M sucrose solution. (B) Photograph of the dechorionated eggs placed and clamped to the dish with tiny nuts. (C) An egg analyzed by ImageJ software; the red line indicates the area defined by related points around the membrane contour of the embryo. Scale bar: 500 μm.

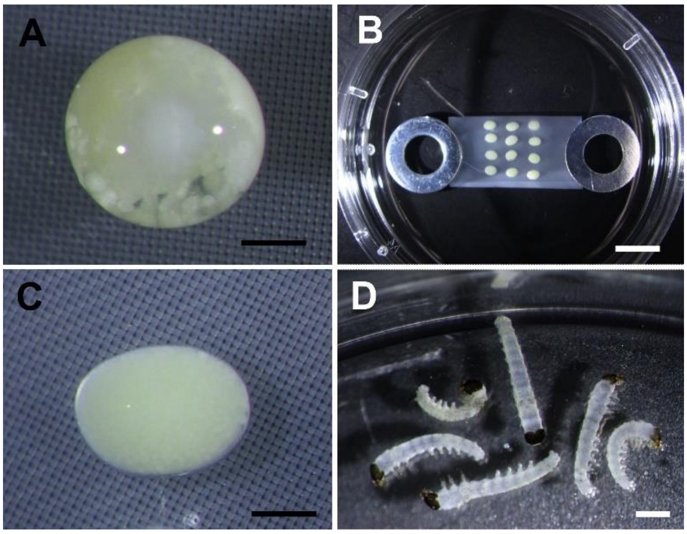


**Figure S2.** Images of permeabilized early embryonic stages of the pnd-w1 strain with a modified culture method using liquid paraffin. (A) An egg with an amorphous shape with the dry-moist culture method. (B) Eggs with the culture method using liquid paraffin. (C) An egg with a normal shape in the culture method using liquid paraffin. (D) Embryos developed following to the serosa ingestion in the modified culture method. Scale bar: 500 μm.

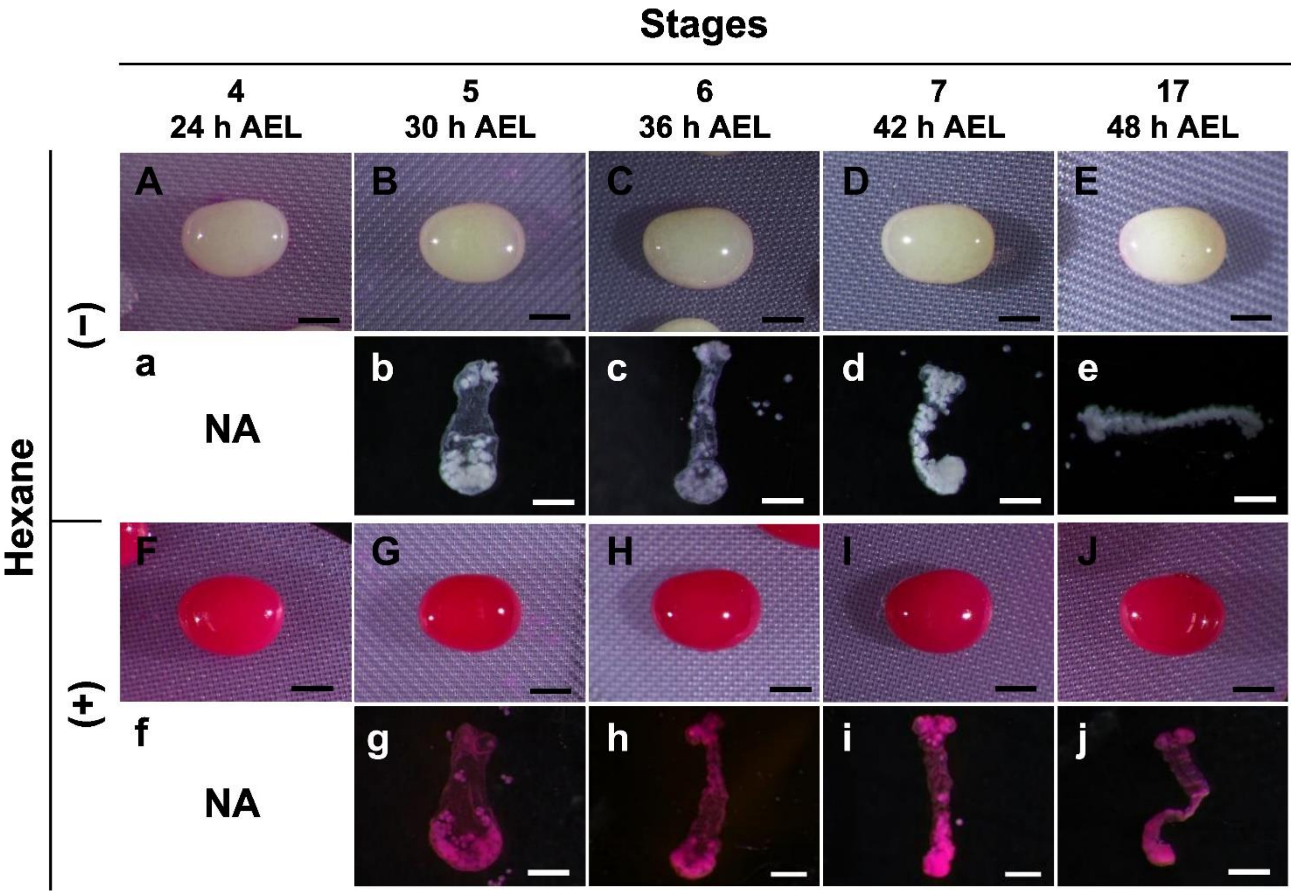


**Figure S3.** Images of permeabilized embryos in the early stages of the pnd-w1 strain. The dechorionated eggs were subjected to a permeabilization treatment with hexane for 30 s, immersed in 0.1% rhodamine B for 10 min, and then rinsed with PBS. (**A-E**) Eggs without hexane treatment. (**a-e**) Naked embryos derived from B-E eggs. (**F-J**) Eggs with hexane treatment. (**f-j**) Naked embryos derived from F-J eggs. Permeabilized embryos were stained red. NA, not available. Scale bar: 500 μm.

**Table S1.** Egg hatchability of fertile moths derived from permeabilized embryos of pnd-w1 and w1 strains.

| Strains | Stage | No. of moths | No. of eggs laid [a] | No. of hatched embryos [b] | Hatchability (%) [c] |
|---|---|---|---|---|---|
| pnd-w1 | Early 1-Stage 25 | 21 | 338.9 ± 23.0 | 278.3 ± 25.2 | 81.9 ± 4.1 |
| w1 | Early 1-Stage 25 | 20 | 379.4 ± 50.3 | 294.5 ± 37.1 | 79.9 ± 4.3 |

[a] Mean number of eggs laid ± SE

[b] Mean number of hatched embryos ± SE

[c] Percentage of hatched eggs to number of eggs laid ± SE
